# Screening Children with a Family History of Central Congenital Hypoventilation Syndrome

**DOI:** 10.1155/2020/2713606

**Published:** 2020-03-26

**Authors:** Hina Emanuel, Kimberly Rennie, Kelly Macdonald, Aravind Yadav, Ricardo A. Mosquera

**Affiliations:** ^1^Department of Pediatrics, The University of Texas Health Science Center at Houston, McGovern Medical School, Houston, TX, USA; ^2^University of Houston, Texas Institute for Measurement, Evaluation, and Statistics, Department of Psychology, Houston, TX, USA

## Abstract

Congenital central hypoventilation syndrome (CCHS) is a rare genetic disorder of an autonomic nervous disorder that affects breathing. It is characterized by respiratory insufficiency secondary to insensitivity to hypoxemia and hypercarbia, particularly during sleep leading to persistent apnea. We report four individuals across two generations harboring heterozygous 25 polyalanine repeats mutations (PARMs) in PHOX2B with a varying degree of phenotypic clinical manifestations. Two family members who reported to be “asymptomatic” were subsequently diagnosed with CCHS, based on genetic testing, obtained because of their family history. Genetic studies in the family including a mother and three offsprings revealed in-frame five amino acid PARMs of PHOX2B consistent with CCHS in addition to full clinical assessment. All affected individuals had evidence of hypercapnia on blood gas analysis with PCO_2_ in the range of 32–70 (mean; 61). Nocturnal polysomnogram revealed evidence of hypoventilation in two individuals (1 offspring and mother) with the end-tidal CO_2_ median of 54. Magnetic resonance imaging of brain revealed no abnormalities in the brain stem. There was no evidence of cor pulmonale on echocardiograms in all individuals. Neuropsychological testing was conducted on all four patients; two patients (mother and 1 offspring) had normal results, while the other two offspring exhibited some impairments on neuropsychological testing. This case series emphasizes the importance of screening first-degree relatives of individuals with confirmed CCHS to minimize complications associated with long-term ventilatory impairment. It also suggests that some patients with CCHS should undergo neuropsychological evaluations to assess for cognitive weaknesses secondary to their CCHS.

## 1. Introduction

Congenital central hypoventilation syndrome (CCHS) is also known as Ondine's curse and is an autosomal dominant disease with an estimated incidence of one per 200,000 live births. CCHS is characterized by respiratory insufficiency, dysregulation of ventilatory hemostasis during nonrapid eye movement (NREM) leading to alveolar hypoventilation, and arterial hypoxemia in settings of normal lung mechanics. CCHS is diagnosed in the absence of primary neuromuscular, metabolic, infectious, pulmonary, or cardiac diseases or brainstem lesion [1]. The disease-defining gene for CCHS is the paired-like homeobox 2B gene (PHOX2B). Mutations in PHOX2B are responsible for CCHS. Polyalanine repeat mutations (PARMs) in PHOX2B account for more than 90% of CCHS cases. The majority of PARMs are considered to arise *de novo*, and about 10% of the mutations are inherited mostly from asymptomatic parents with somatic mosaicism and rarely from affected parents [2,3].

We present the case of a family with a mother and three offsprings from different biological fathers with identical polyalanine repeat mutations of PHOX2B with varying degree of penetrance, expressivity, and, henceforth, clinical manifestations. We highlight discord in phenotypic and genotypic expression among the family of a mother and three offsprings and importance of screening first-degree relatives to identify CCHS in individuals with no overt symptoms.

## 2. Methods

The identification of CCHS in a family of two siblings prompted evaluation of the rest of the family members for CCHS. We performed genetic testing to confirm diagnosis of CCHS in all family members. Individuals with confirmed genetic testing for CCHS underwent complete clinical assessment. Complete clinical assessment included overnight polysomnogram using standard protocol in the mother and two siblings, neuropsychological evaluation using valid standardized scales which was conducted by a licensed neuropsychologist, and a cardiological assessment by using an electrocardiograph and echocardiograph.

**Figure 1:**
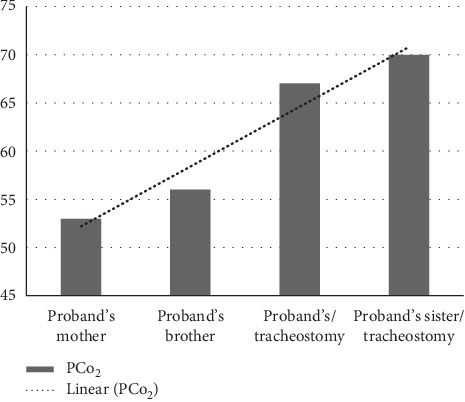
PCO_2_ in index case and first-degree relatives except the father with and without tracheostomy.

All individuals (*n* = 4, age; range, 1–31 years) had PHOX2B sequence analysis carried out, which showed heterozygous p. Ala241 (25) polyalanine repeats, the Epworth Sleepiness Scale (ESS) score between 4 and 5, normal brain MRI, echocardiogram with no cor pulmonale, and full-scale intelligence quotient (FSIQ) and showed average intellectual functioning for proband's brother (106) and proband's mother (94), significant impairment in cognitive, language, and motor development (70) for proband's sister, below average intellectual functioning (83) for proband, and a mean PCO_2_ of 61 mmHg for all four individuals (See Table 1, Figure 1).

## 3. Clinical Details

The proband is a 7-year-old child of Hispanic background who was born at 39 weeks of gestation via normal vaginal spontaneous delivery and had apnea briefly after birth, requiring invasive mechanical ventilation for a week. He was successfully weaned to room air with no recurrence of an apneic event during the neonatal period; however, he was readmitted with acute respiratory failure with hypoxemia and hypercarbia, PCO_2_ max 68 (Table 1, Figure 1), and apneas at five weeks of age, requiring invasive positive pressure ventilation. Infectious, metabolic, cardiac, pulmonary, and brain stem lesion disorders were ruled out. Nocturnal polysomnogram (NPSG) was not performed at the time of diagnosis; however, subsequent polysomnogram performed on positive pressure ventilation (SIMV pressure control; rate 15, pressure control 13, pressure support 15, PEEP 5) revealed an apnea hypopnea index (AHI) of zero per hour, transcutaneous CO_2_ in the range of 30–40's, and average baseline oxygen saturations of 99% on room air (Table 1). Prolonged QT interval was evident on the electrocardiogram. The echocardiogram reported minimal tricuspid regurgitation with no right ventricular strain (Table 1). Chest radiographs reported no lung parenchymal pathology, and magnetic resonance imaging (MRI) ruled out brain stem disorders (Table 1). PHOX2B sequence analysis revealed heterozygous 25 polyalanine repeat mutations which confirmed the diagnosis of CCHS (Table 1).A tracheostomy tube was placed for long-term ventilatory support. He was eventually weaned to room air during a day at one year of age with continued nocturnal ventilatory respiratory support. Neuropsychological testing indicated below-average intellectual functioning. The proband met criteria for a diagnosis of attention deficit hyperactivity disorder (Table 1).

The proband's 12-month-old maternal half-sister was born at 40 weeks of gestation via normal vaginal delivery. She was discharged home on room air 2 days after birth. She was readmitted with multiple episodes of apnea and respiratory failure with hypoxemia and hypercapnia at seven days of life requiring invasive ventilation and eventually tracheostomy placement for long-term ventilatory support. Infectious, cardiological, neurological, and primary pulmonary etiologies were ruled out. No pathological rhythm abnormalities were identified on the electrocardiogram. NPSG was not performed at the time of diagnosis although blood gases showed hypercapnia; PCO_2_ max 70 (Table 1, Figure 1). Echocardiography showed normal heart structures (Table 1). MRI revealed no intracranial or brain stem pathology. Due to high degree of clinical suspicion of CCHS in settings of siblings with CCHS, PHOX2B genetic analysis was sent. PHOX2B sequence analysis revealed heterozygous p. Ala24.1(25) polyalanine repeats which confirmed diagnosis of CCHS (Table 1). Neuropsychological testing for this patient indicated significant impairment in her cognitive, language, and motor development (Table 1). Screening for socioemotional and behavioral disorders was negative.

The case of two siblings with CCHS prompted screening of other family members, which included the mother and the 10-year-old brother. In addition to routine screening as part of standard care of CCHS, we included clinical assessment to inquire about the symptoms of CO_2_ narcosis, autonomic dysregulation, day-time sleepiness, anxiety, and depression. There was no evidence of clinical manifestations of autonomic dysregulation in other family members. The proband's 10-year-old full brother was born at 36 weeks of gestation via normal vaginal delivery with a medical history significant for episodes of apnea in settings of respiratory syncytial virus but otherwise have been unremarkable from pulmonary standpoint. PHOX2B analysis was performed at 10 years of age for screening in settings of the family history of CCHS. PHOX2B sequence analysis showed identical *PHOX2B* mutation 25 polyalanine repeats (Table 1). Blood gas showed a PCO_2_ of 58 (Table 1, Figure 1). NPSG revealed an apnea hypopnea index (AHI) of 3.4/hr, obstructive AHI of 3.4/hr, central AHI of 0.2/hr, and REM supine AHI of 0.2/hr (Table 1). End-tidal CO_2_ averaged 56 during NREM sleep, with a maximum value of 66, and remained above 50, 79% of total sleep time (TST). Average oxygen saturation remained 94% during TST (Table 1). EES was reported as 4/24 (Table 1). The echocardiogram showed small PDA, trivial pulmonary, and tricuspid regurgitation with no right ventricular strain. No pathological rhythm variants on the electrocardiogram were identified. MRI of the brain revealed no evidence of brain stem disorders. No ophthalmological, neurological, or gastrointestinal problems were observed. Neuropsychological testing indicated intact intellectual functioning, and no concerns regarding socioemotional and behavioral functioning were identified (Table 1).

The proband's mother had PHOX2B sequence analysis carried out at thirty years of age. Results of PHOX2B analysis showed heterozygous p. Ala241(25) polyalanine repeats (Table 1). She reported early morning headaches and fatigue upon awakening. She did not have cardiological, ophthalmological, and neurological evaluation performed at the time of this writing. A blood PCO_2_ of 54 was observed (Table 1, Figure 1). Nocturnal polysomnogram showed a total sleep efficiency of 87% with a TST of 400 mins, AHI index of 12.6/hr, recorded average oxygen saturation of 94%, and end-tidal CO_2_ averaged 52 torr during entire sleep and >50 torr for 60% of TST. The ESS score was reported as 5/24 (Table 1). Results of the mother's neuropsychological testing indicated intact intellectual functioning (Table 1).

## 4. Discussion

This family demonstrates a novel *PHOX2B* p. Ala24.1(25) gene in four individuals across two generations with highly variable penetrance ranging from respiratory failure during the neonatal period to later age with hypoventilation while asleep. Our study emphasizes the importance of screening of parents and at-risk siblings with subtle clinical findings through genetic analysis to identify PHOX2B pathogenic variant. In addition, given the rarity of disease, varied clinical manifestation, and lack of experience of medical professionals as evidenced by the previous literature, this study seeks to increase awareness of CCHS diagnosis, clinical manifestation, and long-term outcomes [4,5]. Through incorporation of questions pertaining to sleep, neurocognitive functioning, and general well-being, this study highlights the importance of comprehensive evaluation of CCHS including NPSG, evaluation of breathing while awake, echocardiogram, Holter monitoring and screening for neurocristopathies, and neuropsychological evaluation. Our case study highlights the importance of screening of at-risk asymptomatic family members through screening questions and a pertinent medical history followed by genetic testing. Genetic testing should not be limited to parents alone but should be extended to all family members at risk for CCHS. Had it not been for screening, the proband's 10-year-old brother and the mother would have remained undetected.

A mutation in the *PHOX2B* gene is a requisite to diagnosis of CCHS. PHOX2B is involved in the regulation of the autonomic nervous system and respiratory control neurons. The majority (>90%) of PHOX2B mutations are heterozygous for an in-frame triplet duplication PARMs with the resultant genotype of 24–33 alanine repeats [6]. The CCHS phenotype has not been associated with any degree of somatic mosaicism so far, suggesting a germline origin for most PARMs in affected CCHS patients, which is consistent with our case series. De novo PHOX2B pathogenic variant accounts for most of the cases of CCHS with remaining cases of CCHS transmitted from affected parents with some degree of mosaicism, germline or somatic in PHOX2B variant with 50%, or a lower chance of acquiring pathogenic variant. The presence of the same PHOX2B mutation in parent-offspring supports an autosomal mode of inheritance. Our case series presents the identical PHOX2B mutation with varying degrees of penetrance and the time of onset which could be secondary to gene modifiers [7,8].

The evaluation of parents, children, and at-risks siblings of individuals with CCHS depends on the pathogenic variant identified in the proband. Our study emphasizes the importance of screening first-degree relatives of an individual in a stepwise approach [9–11], prenatal testing, and genetic counseling to make informed medical and personal decisions. The PHOX2B screening test (fragment analysis) is performed if child has PARM or frame shift NPARM. PHOX2B mutation confirmed CCHS. If no mutation is identified, no further testing is advised; however, germline mosaicism cannot be ruled out. If the PHOX2B pathogenic variant is identified, there is a risk of transmitting mutation in pregnancy and warrants prenatal testing as the risk to the sibs is 50% if the proband's parent is affected, risk is 50% or lower if the proband's affected parent has mosaicism for the PHOX2 pathogenic variant, and even if the proband's parents are unaffected, there still remains risk to the proband's siblings due to mosaicism. Once the pathogenic variant is identified, genetic counseling can be offered to individuals at risk for CCHS to make informed decisions, prenatal testing, and preimplantation genetic diagnosis and to discuss future outcomes for themselves and offsprings before pregnancy. Results of our neuropsychological testing suggest that cognitive deficits may be associated with medical severity of CCHS. Routine neuropsychological testing may be warranted, especially for patients with more severe forms of the disease.

In conclusion, this case series highlights the importance of screening at-risk family members of an individual with CCHS; so genetic counseling can be offered to at-risk individuals in regard to long-term health implications of CCHS. This case report emphasizes the need of validated tools to screen for specific sleep, neurocognitive, and general well-being questions pertaining to CCHS in family members of the affected individual. Further research is needed to provide insight into applicability and outcomes of screening tools in clinical practice. With early diagnosis and careful ventilatory management, the sequelae of hypoxia and morbidity should be minimized and long-term outcome improved.

## Figures and Tables

**Table 1:** Baseline characteristics of the study population.

Case	Age (years)	PHOX2B	PCO_2_ (maximum)	ESS^*∗*^	AHI/hr^*∗∗*^	O_2_ sat^*∗∗∗*^ (average)	MRI brain	ECHO (cor pulmonale)	FSIQ^*∗∗∗∗∗∗*^
Proband	7	p. Ala24.1(25)	67	4	0^*∗∗∗∗*^	99%	Normal	Negative	83
Proband's sister	1	p. Ala24.1(25)	70	N/A^*∗∗∗∗∗*^	N/A	97%	Normal	Negative	70
Proband's brother	10	p. Ala24.1(25)	56	4	3.4	94%	Normal	Negative	106
Proband's mother	31	p. Ala24.1(25)	53	5	12.6	94%	N/A	Negative	94

^*∗*^Epworth sleepiness scale. ^*∗∗*^Apnea hypopnea index. ^*∗∗∗*^Oxygen saturation on room air. ^*∗∗∗∗*^NPSG performed on respiratory support with resultant AHI of 0/hr. ^*∗∗∗∗∗*^Data not available at time of this writing. ^*∗∗∗∗∗∗*^Full-scale IQ as measured by the Wechsler scale of intelligence for children-fifth edition for the proband and the proband's brother and the Wechsler adult intelligence scale of intelligence-fourth edition for the proband's mother. For the proband's sister, the score reflects the patient's performance on the cognitive development portion of the Bayley scales of infant development-third edition. Scores falling between 85 and 115 are within the normal limits.
